# High Diagnostic Yield of Endoscopic Retrograde Cholangiopancreatography Brush Cytology for Indeterminate Strictures

**DOI:** 10.1093/jcag/gwac011

**Published:** 2022-05-05

**Authors:** Abdulsemed M Nur, Misbah Salim, Scott Boerner, Suqing Li, Cindy C Y Law, Leanne Edwards, Kaitlin Ryan, Paul D James

**Affiliations:** Department of Medicine, University Health Network, University of Toronto, Toronto, Canada; Department of Laboratory Medicine and Pathobiology, University Health Network, University of Toronto, Toronto, Canada; Department of Medicine, University Health Network, University of Toronto, Toronto, Canada; Department of Medicine, University Health Network, University of Toronto, Toronto, Canada; Department of Medicine, University Health Network, University of Toronto, Toronto, Canada; Department of Medicine, University Health Network, University of Toronto, Toronto, Canada; Department of Medicine, University Health Network, University of Toronto, Toronto, Canada; Department of Medicine, University Health Network, University of Toronto, Toronto, Canada

**Keywords:** *Biliary*, *Endoscopic retrograde cholangiopancreatography*, *Malignancy*, *Stricture*

## Abstract

**Background:**

Endoscopic retrograde cholangiopancreatography (ERCP) brush cytology is used frequently for sampling indeterminate biliary strictures. Studies have demonstrated that the diagnostic yield of brush cytology for malignant strictures is estimated to be 6%–70%. With improved diagnostic tools, sampling techniques and specimen processing, the yield of ERCP brush cytology may be higher. This study aimed to assess the yield of brush cytology and determine factors associated with a positive diagnosis.

**Methods:**

This was a cohort study of patients who underwent ERCP brush cytology from October 2017 to May 2020. Patient demographics, clinical, procedural and pathological data were collected using chart review. Sampling data were captured up to 3 months post-index ERCP to capture repeat brushings, biopsies or surgical resections. Outcomes included the diagnostic yield, true/false positive values and true/false negative values of malignancy detection using ERCP brush cytology.

**Results:**

A total of 126 patients underwent a brush cytology, 58% were male and 79% had a stricture in the extrahepatic region. Ninety-three patients were diagnosed with a malignancy, of which 78 had positive brush cytology results and 15 had a negative brush cytology result. The diagnostic yield, sensitivity, specificity, positive predictive value, negative predictive value and accuracy were 84%, 83%, 97%, 99%, 68% and 87% respectively.

**Conclusion:**

ERCP brush cytology performed using updated sampling technique is associated with high diagnostic yield. This allows for earlier malignancy diagnosis, timely treatment and decreased need for further investigation.

## INTRODUCTION

The management and outcomes related to biliary stricture are highly dependent on the nature of the stricture. Benign strictures are usually treated with endoscopic dilation and have a very favorable prognosis. On the other hand, malignant strictures are associated with high morbidity and mortality and are treated with stenting or surgical resection. Early detection and accurate pathology-confirmed diagnosis are crucial as they can impact patients’ surgical candidacy as well as eligibility for potential targeted therapies (1–5).

In this study, an indeterminate stricture was defined as any stricture without definite diagnosis after cross-sectional imaging and that require further investigation to rule out malignancy. The gold standard for the diagnosis of malignancy in a biliary stricture is to perform surgical resection. While this provides a definitive diagnosis, surgery is associated with significant morbidity and up to 30% of biliary strictures are benign. Hence, a high preoperative probability of malignancy is essential. Other widely used methods of evaluating biliary strictures include radiological tests and percutaneous tissue sampling. Imaging modalities such as abdominal ultrasonography, computed tomography and magnetic resonance imaging are useful in identifying the presence of a biliary stricture or related mass lesion. Unfortunately, these tests are unable to provide a tissue diagnosis. On the other hand, percutaneous tissue sampling can provide tissues specimens but is invasive, complicates the surgical field and carries a risk of tumor seeding (6, 7).

Technological advances have led to the emergence of a number of endoscopic methods of evaluating biliary strictures. These include endoscopic ultrasonography-guided fine needle biopsy (EUS-FNB), cholangioscopy-guided forceps biopsy and endoscopic retrograde cholangiopancreatography (ERCP) brush cytology (8–10). Of these options, ERCP brush cytology is the most widely available and commonly used. To date, however, the reported diagnostic yield of this technique ranges from 6% to 70% (11–13). In a recent meta-analysis of 9 studies, the pooled sensitivity and specificity of brush cytology for the diagnosis of malignant biliary strictures were 45% and 99%, respectively (14). Multiple techniques have been proposed to increase the yield of brushing samples, including stricture dilatation, repeat brushing and using modified cytology brushes. In addition to these novel techniques, advances in cytological processing and assessment may further increase the diagnostic sensitivity of brush cytology (15–19).

This aim of this study is to determine the accuracy of updated brush sampling and cytology techniques in patients with indeterminate biliary strictures and to identify factors that influence the diagnostic yield.

## MATERIAL AND METHODS

### Study Population and Data Collection

This is a single-centre retrospective study of all patients who underwent an ERCP procedure with brush cytology at UHN from October 1, 2017 to May 30, 2020. Patients were identified using the UHN operating room scheduling database, which records patient demographics and procedure details. Patient demographics, clinical, procedural and pathological data were also collected via chart review. Patients’ clinical information was available to the physician who performed the ERCP as well as the cytopathology team that analyzed the ERCP brushing samples. The results of the gold standard tests (diagnosis of malignancy by ERCP, EUS-FNB, percutaneous biopsy and or surgical resection) were not available to them at the time of the ERCP or analysis of brushings. Clinical information and index test results were available to the assessors of the gold standard tests.

### ERCP Brushing Technique

**Figure 1.**
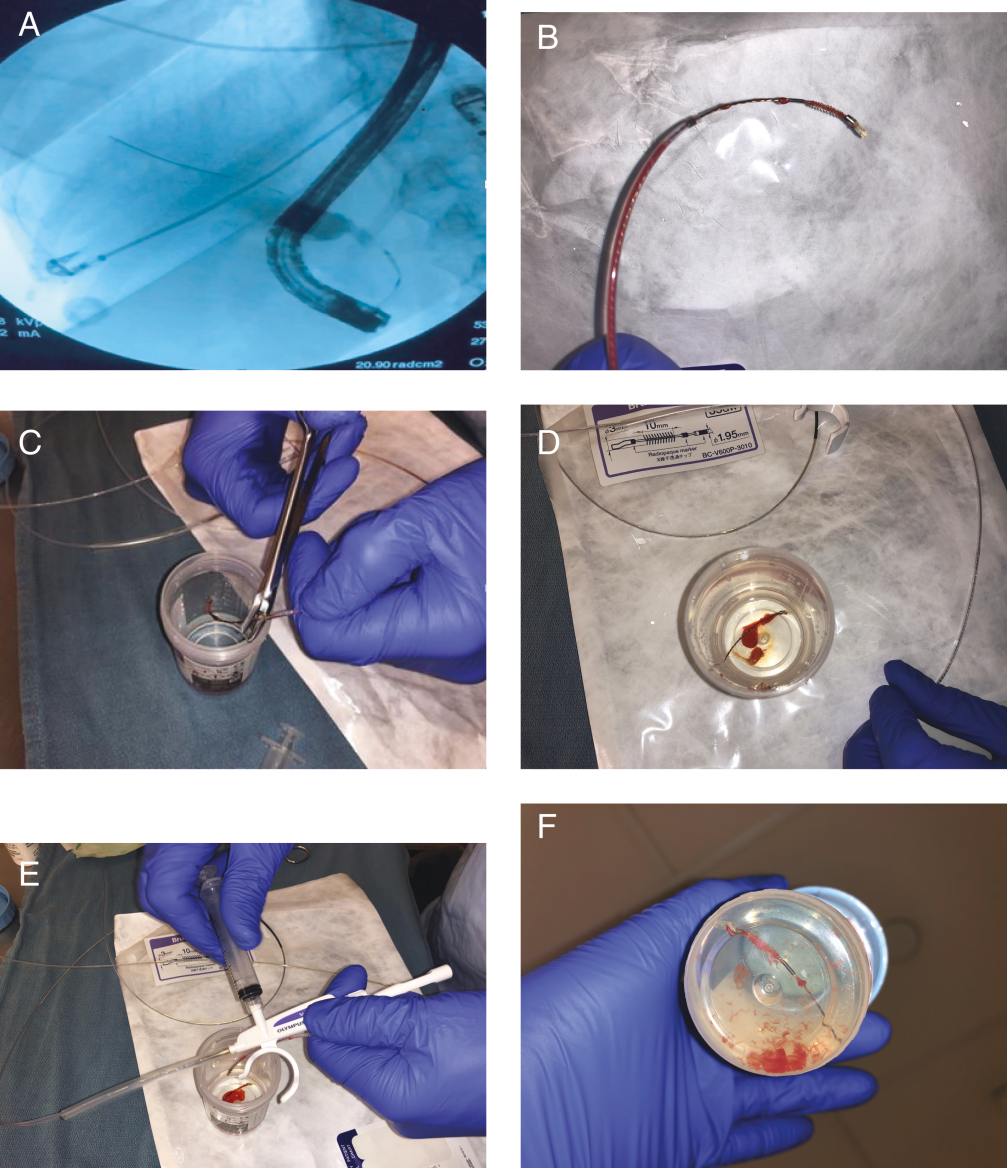
ERCP brushings technique and sample processing. The cytology brush was advanced through the entire stricture in a to-and-fro fashion ten times under direct fluoroscopic guidance (a). The catheter was then removed (b). The cytology brush was then cut at the wire using surgical scissors (c) and placed into a methanol based buffered solution (CytoLyt) (d). 2–3 mL of the solution was then aspirated into a 10-mL syringe and used to flush residual sample from the catheter sheath into the sample container (e and f).

All procedures were performed directly by or under supervision of one endoscopist (P.J.), who used the same technique of acquiring and processing biliary brushing samples in all patients. The ERCP brushing samples were obtained according to the following procedure: After cannulation of the common bile duct and biliary stricture characterization by cholangiography, we ensured that the guidewire crossed through the stricture being sampled. A sphincterotomy was then performed and the sphincterotome was removed while leaving the guidewire in place. A double-lumen wire-guided cytology brush catheter was then introduced into the biliary system and advanced to the distal aspect of the stricture. The cytology brush was advanced through the entire stricture in a to-and-fro fashion ten times under direct fluoroscopic guidance (Figure 1a). The catheter was then removed (Figure 1b). The cytology brush was then cut at the wire using surgical scissors (Figure 1c) and placed into a methanol-based buffered solution (CytoLyt) (Figure 1d). Two to three milliliters of the solution was then aspirated into a 10-mL syringe and used to flush residual sample from the catheter sheath into the sample container (Figure 1e and f).

### ERCP Brushing Sample Processing and Evaluation

One alcohol fixed, Papanicolaou stained ThinPrep slide was produced from each brushing sample. A paraffin block was produced from the remaining 1 mL of the residual sample followed by fixation with 10% neutral buffered formalin. All ERCP brushing samples were evaluated by cytopathologists at UHN.

### Outcomes

Outcomes examined include the diagnostic yield, true positive, false positive, true negative and false negative values of malignancy detection using ERCP brush cytology. All patients were followed for a minimum of 3 months after their index ERCP and any post-ERCP sampling via repeat ERCP brushings, EUS-FNB, percutaneous biopsy or surgical resection was recorded.

Patients were considered true positives for malignancy if the index ERCP brushing cytopathology report noted ‘adenocarcinoma’ or ‘suspicious for malignancy’ and the diagnosis was either confirmed by further investigations. or no further investigations were performed. At our centre, further sampling is not performed after the majority of positive brushing cytology results as it is concluded that a diagnosis has been made. Patients with ERCP brushing negative for malignancy were followed for at least 3 months to determine if follow-up testing led to a diagnosis of malignancy. Results from follow-up procedures (repeat ERCP, EUS-FNB, percutaneous biopsy or surgical resection) were used to derive the diagnostic rate (proportion of malignant cases that had positive ERCP brush cytology). The gold standard was the diagnosis of malignancy by ERCP, EUS-FNB, percutaneous biopsy and/or surgical resection.

The relationship between selected clinical and biochemical variables with diagnostic yield was also examined. These factors were determined *a priori* and include patient age, sex, stricture location, diagnosis of primary sclerosing cholangitis (PSC), alanine transaminase (ALT), alkaline phosphatase level (ALP), bilirubin level, gamma-glutamyl transferase level (GGT), carbohydrate antigen 19-9 level (CA 19-9) and IgG4 level. For the purposes of this study, proximal strictures include intrahepatic and hilar strictures. All strictures distal to the hilum were noted to be distal.

### Statistical Analysis

Sensitivity, specificity, positive predictive value and negative predictive values for brush cytology were calculated. Descriptive trends as well as associations were evaluated. Categorical variables were presented as proportions and continuous variables using median and interquartile range. Two-sided student t-testing was used to compare the means of normally distributed continuous variables and the Mann–Whitney U test was used to compare medians of continuous variables that were non-normally distributed. The chi-squared test was used to compare categorical variables and Fisher’s exact test was reserved for cases where there were fewer than 5 observed events. Univariate logistic regression was used to evaluate potential predictors of the primary outcome. The multivariate regression model was adjusted for age, sex, stricture location, diagnosis of PSC, ALT, ALP, bilirubin, GGT, CA 19-9 and IgG4 level. A *P* value of ≤0.05 was considered to indicate statistical significance and all 95% confidence intervals were two-sided. Statistical analysis was conducted using STATA version 13.1 (*Stata Statistical Software: Release 13*. College Station, TX).

## RESULTS

**Figure 2.**
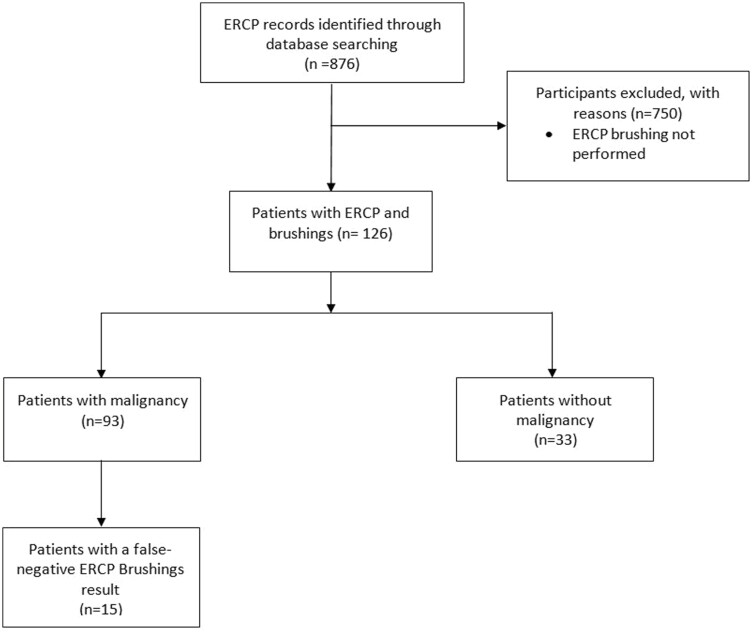
Patient flowchart.

Baseline patient characteristics are outlined in Table 1. A total of 126 patients underwent an ERCP brush cytology procedures during the study period (Figure 2), of which 58% were male. Ten patients (8%) had a diagnosis of PSC and 99 patients (79%) had a stricture in the extrahepatic region.

**Table 1.** Patient baseline characteristics and ERCP brush cytology results among patients with malignancy

	All (*n* = 126)	Patients with malignancy (*n* = 93)		
		ERCP brush cytology positive (*n* = 78)	ERCP brush cytology negative (*n* = 15)	*P* value^1^
Age, mean (SD)	66 (15)	70 (14)	69 (13)	0.8
Sex				
Male	73 (58)	43 (55)	7 (47)	0.4
Female	53 (42)	35 (45)	8 (53)	
Primary sclerosing cholangitis				0.6
Yes	10 (8)	3 (4)	–	
No	116 (92)	75 (96)	15 (100)	
Stricture Location				
Proximal	27 (21)	15 (19)	2 (13)	0.3
Distal	99 (79)	63 (80)	13 (86)	
ALT (median, IQR)	55.5 (60)	40 (50)	50 (39)	0.1
ALP (median, IQR)	441 (475)	526 (496)	484 (789)	0.7
BILI (median, IQR)	53.5 (55)	41 (43)	33 (46)	0.4
GGT (median, IQR)	35 (13)	20 (2)	20 (11)	0.3
CA 19-9 (median, IQR)	52 (56)	40 (43)	46 (42)	0.8
IgG4 (median, IQR)	23 (2.5)	13 (0)	13 (0)	0.1

SD, standard deviation; IQR, interquartile range; ALT, alanine transaminase; ALP, alkaline phosphatase level; BILI, bilirubin; GGT, gamma-glutamyl transferase; CA 19-9, carbohydrate antigen 19-9.

Two-sided student t-testing was used to compare normally distributed continuous variables and the Mann–Whitney U test was used to compare non-normally distributed continuous variables. Chi-squared test was used to compare categorical variables and Fisher’s exact test was used when there were fewer than 5 observed events. A *P* value of ≤0.05 was considered statistically significant.

Overall, 93 patients were diagnosed with a malignancy. Of these, 78 had positive brush cytology results, 15 had negative brush cytology results, and patient characteristics did not differ significantly between these two groups. The diagnostic yield, sensitivity, specificity, positive predictive value, negative predictive value and accuracy were 84%, 83%, 97%, 99%, 68% and 87%, respectively. Diagnostic yields were similar in patient subgroups based on gender (male 86%, female 81%), stricture location (proximal 88%, distal 83%) and diagnosis of PSC (PSC 100%, no PSC 83%).

Factors associated with positive brush cytology results are outlined in Table 2. The odds of a positive brush cytology result increased by 3% for each additional year of age (*P* = 0.002; 95% CI 1.00–1.06). However, this finding did not remain significant in the fully adjusted model. Elevated ALP was associated with slightly increased odds of positive brush cytology (OR 1.00; 95% CI 1.00–1.01), but no association was observed following adjustment. Patient gender, stricture location, ALT, bilirubin, GGT, CA 19-9, and IgG4 levels did not appear to significantly impact the odds of a positive brush cytology result. These findings remained consistent in both the unadjusted and adjusted models.

**Table 2.** Odds of positive ERCP brush cytology among patients with malignancy by patient and stricture factors

	Unadjusted odds ratio (*n* = 126) (95% CI)	Adjusted odds ratio (*n* = 126) (95% CI)
Age, mean (SD)	1.03 (1.00–1.06)*	1.03 (0.99–1.06)
Sex		
Male	REF	REF
Female	1.28 (0.6–2.7)	2.56 (0.9–7.3)
Diagnosis		
Primary sclerosing cholangitis	0.36 (0.1–1.4)	0.67 (0.09–4.8)
Other	REF	REF
Stricture Location		
Proximal	0.83 (0.34–1.9)	0.5 (0.14–1.7)
Distal	REF	REF
ALT	0.99 (0.98–1.00)	0.99 (0.97–1.00)
ALP	1.00 (1.00–1.01)*	1.00 (1.00–1.00)*
BILI	1.00 (0.98–1.01)	1.00 (0.98–1.01)
GGT	1.03 (0.99–1.07)	1.03 (0.98–1.01)
CA 19-9	1.00 (0.98–1.01)	0.99 (0.97–1.00)
IgG4	1.04 (0.98–1.11)	1.06 (0.98–1.15)

SD, standard deviation; IQR, interquartile range; ALT, alanine transaminase; ALP, alkaline phosphatase level; BILI, bilirubin; GGT, gamma-glutamyl transferase; CA 19-9, carbohydrate antigen 19-9.

## DISCUSSION

Using updated sampling and processing techniques, we report a diagnostic rate of 83% and accuracy of 87% using ERCP brushings. The specificity, positive predictive value and negative predictive value of biliary brush cytology in our patients were 97%, 99%, and 68%, respectively. This is one of the highest diagnostic yields from this method reported in the literature to date.

Obtaining timely tissue diagnosis of indeterminate strictures is crucial for a number of reasons. As most patients present with advanced disease and there is high mortality associated with biliary malignancy, efficient and reliable diagnostic methods facilitate swift initiation of therapy (20). Moreover, increased sensitivity of ERCP brush cytology would decrease the need for additional investigations such as EUS-FNB, repeat ERCP, cholangioscopy and surgical exploration. While these are all valuable tools, it is ideal to avoid performing multiple procedures that may be more invasive and place the patient at an increased risk of complications such as bleeding, perforation and acute pancreatitis (21, 22). It is also important to consider the financial and psychological toll of multiple hospital visits and investigations on patients and their caregivers. From a healthcare systems perspective, performing multiple procedures results in increased costs and consumption of material and human resources. Finally, some technologies such as cholangioscopy requires additional expertise and may not be widely available.

Our findings are in contrast to previous studies that have reported a modest diagnostic sensitivity of ERCP brushings (2, 9, 11, 23). A meta-analysis involving 16 studies and 1556 patients by Burnett et al. suggested an overall sensitivity of 42% (24). Studies by Harewood et al. and Draganov et al. reported diagnostic sensitivities of 18% and 5.9%, respectively (9, 23). On the other hand, sensitivities greater than 60% have been reported by Arvanitakis et al. and Urbano et al. (25, 26). Factors that have been hypothesized to explain this wide variation in sensitivity include differences in sampling technique, type of brush, use stricture dilation followed by brush sampling, cytological sampling techniques, inter-pathologist variation in interpretation, location of the tumor and patient population (2, 11, 12, 23, 24, 27–29).

We believe that the diagnostic yield of ERCP cytology brushings at UHN is higher than reported in the literature for multiple reasons. Firstly, we use an updated technique that is inspired by EUS-FNA principles. This includes directly visualizing the stricture under fluoroscopy and brushing the strictures to-and-fro 10 times. Traditionally, endoscopists brush strictures with fewer passes and without direct fluoroscopic visualization. Secondly, we also take additional steps to ensure that all of the tissue collected by the brush is sent for cytology. This includes cutting off the brush, submerging it in Cytolyt and flushing residual specimen out of the catheter. Lastly, all samples are sent to a cytopathology department which is highly experienced in the examination of biliary cytology.

Modified techniques have been described in the past to improve the yield of brush cytology. Increased exfoliation of tumor cells due to stricture manipulation has been reported to increase the diagnostic yield of biliary brush cytology (18, 29, 30). Farrell et al. reported that stricture dilatation to 10 French and endoscopic needle aspiration significantly increased the sensitivity and specificity of biliary brush cytology (18).

Additionally, a randomized trial reported the use of a dedicated basket to be associated with increased sensitivity compared to conventional brush cytology (30).

Patient demographics, stricture location and biochemical parameters did not significantly impact the odds of obtaining a positive brush cytology result. This is contrast to several studies on this topic. Mahmoudi et al. reviewed 189 patients and reported that a positive yield was associated with older age, a mass size >1 cm and a stricture length of >1 cm (2). Witt et al. found that the risk of malignancy was associated with age, stricture in the distal CBD, endoscopic/fluoroscopic appearance suspicious for malignancy, presence of a pancreatic head mass and CA 19-9 level >300 IU (31). Additionally, Park et al. reported that elevated levels of CA19-9, CEA, ALP, and GGT and stricture length were associated with malignant strictures in patients with indeterminate biliary strictures on imaging and atypical or suspicious cells on brush cytology (32). The reason for the differences between our findings and the above studies may be due to our observed higher diagnostic yield. Realizing a higher overall diagnostic yield may reduce the influence of these factors in predicting a positive brush cytology result.

Several limitations to our study should be acknowledged. This study included a modest number of patients, which may have limited statistical power. However, the number of patients in this study is similar in size to other studies in this area (2, 31, 32). Secondly, the study had a limited follow-up period of 3 months. On the other hand, the survival of patients with cholangiocarcinoma is limited and the probability of reaching a diagnosis within 3 months of presentation is high because the malignancy progresses rapidly. Of note, no patients in this study were lost to follow up. Thirdly, there is the possibility of referral bias. The impact of this was minimized as we examined the diagnostic yield only among cases confirmed to be malignant. Finally, as with all retrospective studies, the impact of unmeasured confounders cannot be excluded.

## CONCLUSION

In conclusion, using updated sampling and specimen processing techniques, as well as specialized cytological evaluation can substantially improve the yield of ERCP biliary brush cytology. This decreases the need for further invasive investigations as well as reduces the costs and potential risks to patients associated with these procedures. Brush cytology is an efficacious, safe and cost-effective method of assessing biliary strictures and should be considered the initial diagnostic modality in the evaluation of biliary strictures. Larger, prospective studies applying the tools and techniques described in this study are required to confirm our findings.
